# The impact of *PIK3CA* mutations and PTEN expression on the effect of neoadjuvant therapy for postmenopausal luminal breast cancer patients

**DOI:** 10.1186/s12885-023-10853-y

**Published:** 2023-04-27

**Authors:** Shouko Hayama, Rikiya Nakamura, Takayuki Ishige, Takafumi Sangai, Masahiro Sakakibara, Hiroshi Fujimoto, Emi Ishigami, Takahito Masuda, Ayako Nakagawa, Ryotaro Teranaka, Satoshi Ota, Sakae Itoga, Naohito Yamamoto, Takeshi Nagashima, Masayuki Otsuka

**Affiliations:** 1 Department of General Surgery, Graduate School of Medicine, Chiba University, 1-8-1 Inohana, Chuo-Ku, Chiba-Shi, Chiba, 260-8677 Japan; 2 Department of Breast Surgery, Chiba Cancer Center, 666-2 Nitona, Chuo-Ku, Chiba-Shi, Chiba, 260-8717 Japan; 3 Department of Molecular Diagnosis, Graduate School of Medicine, Chiba University, 1-8-1 Inohana, Chuo-Ku, Chiba-Shi, Chiba, 260-8677 Japan; 4 Department of Breast and Thyroid Surgery, Kitasato University School of Medicine, 1-15-1 Kitazato Minami-Ku, Sagamihara, Kanagawa 252-0374 Japan; 5 Departments of Breast Surgery, Toho University Sakura Medical Center, 564-1 Shimoshizu, Sakura, Chiba, 285-8741 Japan; 6 Department of Pathology, Chiba University Hospital, 1-8-1 Inohana, Chuo-Ku, Chiba-Shi, Chiba, 260-8677 Japan; 7 Department of Applied Genomics, Kazusa DNA Research Institute, 2-6-7 Kazusa-Kamatari, Kisarazu Chiba, 292-0818 Japan

**Keywords:** Breast neoplasms, Phosphatidylinositol 3-Kinases/genetics, PTEN Phosphohydrolase, Neoadjuvant therapy, Prognosis

## Abstract

**Background:**

There is pressing needs to find the biomarker in the selection of neoadjuvant therapy in postmenopausal luminal breast cancer patients. We examined the hypothesis that *PIK3CA* mutations and low phosphatase and tensin homolog (PTEN) expression affect the response to neoadjuvant therapy and prognosis in postmenopausal luminal breast cancer patients.

**Methods:**

Postmenopausal patients with estrogen receptor-positive, human epidermal growth factor receptor 2-negative breast cancer, up to stage II, who underwent neoadjuvant chemotherapy (NAC; *n* = 60) or neoadjuvant endocrine therapy (NAE; *n* = 55) were selected. *PIK3CA* exon 9 and exon 20 mutations were screened by high resolution melting analysis and confirmed by Sanger sequence. PTEN expression was evaluated by immunohistochemistry. The relationships among *PIK3CA* mutations, PTEN expression, clinicopathological features, the pathological effect of neoadjuvant therapy, recurrence-free survival (RFS) and overall survival were analyzed.

**Results:**

Among 115 patients, *PIK3CA* mutations and low PTEN expression before treatment were detected in 35 patients (30.4%) and in 28 patients (24.3%), respectively. In the NAC group, tumor with *PIK3CA* mutations showed significantly poorer response than tumor with *PIK3CA* wild-type (*p* = 0.03). On the other hand, in the NAE group, there was no significant difference in pathological therapeutic effect between tumor with *PIK3CA* mutations and tumor with *PIK3CA* wild-type (*p* = 0.54). In the NAC group, the log-rank test showed no difference in RFS between patients with *PIK3CA* mutations and *PIK3CA* wild-type (*p* = 0.43), but patients with low PTEN expression showed significantly worse RFS compared to patients with high PTEN expression (5 year RFS 0.64 vs. 0.87, *p* = 0.01). In the Cox proportional hazards model for RFS, PTEN expression, progesterone receptor, and pathological therapeutic effect were predictive factors for time to recurrence (All *p* < 0.05).

**Conclusions:**

*PIK3CA* mutations are associated with resistance to NAC but do not affect the response to NAE. Low PTEN expression does not affect response to either NAC or NAE but correlates with shorter RFS in patients who received NAC. These biomarkers will be further evaluated for clinical use to treat postmenopausal luminal breast cancer patients.

**Supplementary Information:**

The online version contains supplementary material available at 10.1186/s12885-023-10853-y.

## Background

Luminal breast cancer is the most frequent intrinsic subtype, accounting for about 70% of breast cancers [1]. Patients with luminal breast cancer have treatment options including chemotherapy and endocrine therapy; however, methods for optimizing remain unclear. Comprehensive genetic analysis reveals genetic heterogeneity of breast cancer [1, 2]. Several genetic alterations are frequently observed among breast cancers. These are correlated with intrinsic subtype. Recently, these genetic characteristics were found to be associated with therapeutic efficacy and prognosis [3, 4]*. PIK3CA* gene encoding the p110 catalytic subunit of PI3K is most frequently mutated in luminal breast cancer [1]. *PIK3CA* mutations cause activation of PI3K. PI3K converts phosphatidylinositol bisphosphate (PIP2) into phosphatidylinositol triphosphate (PIP3) and activates Akt. Phosphatase and tensin homolog (PTEN) dephosphorylates PIP3 to PIP2 and regulates Akt signaling [5]. PTEN is often deregulated in patients with breast cancer and can activate PI3K/Akt signaling [1]. Akt acts downstream of PI3K to regulate many biological processes, such as cell proliferation, metabolism, differentiation, apoptosis and tumorigenesis [6]. In patients with human epidermal growth factor receptor 2 (HER2) overexpressed breast cancer, *PIK3CA* mutations and PTEN loss have been reported to cause activation of the downstream PI3K / Akt pathway associated with drug resistance [5, 7]. Moreover, activation of the PI3K / Akt signaling is associated with endocrine therapy resistance [8, 9]. It would be important to investigate how *PIK3CA* mutations and PTEN loss, key genetic alterations in this drug resistance-associated pathway, relate to response to preoperative therapy in early breast cancer.

There is pressing needs to find the biomarker in the selection of neoadjuvant therapy in postmenopausal luminal breast cancer patients. Immunochemical markers such as Ki67, morphological markers such as tumor-infiltrating lymphocytes (TIL), and genomic profile markers such as the Oncotype DX Recurrence Score have been used in studies, but there is insufficient evidence to support their use to guide clinical decisions for neoadjuvant therapy [10]. Several retrospective studies have reported that patients with *PIK3CA* mutation or PTEN loss are less responsive to preoperative chemotherapy in all subtypes, as well as in HER2-positive and triple-negative breast cancer [11–13]. However, this has not been well investigated in luminal breast cancer. We examined the hypothesis that *PIK3CA* mutations and low PTEN expression affect the response to neoadjuvant therapy and prognosis in postmenopausal luminal breast cancer patients. First, we investigated the correlation between the pathological effect of neoadjuvant therapy and *PIK3CA* mutations or low PTEN expression. Second, we assessed these alterations on prognosis using the same cohort.

## Methods

### Patients and clinicopathological data

We selected patients with Estrogen receptor (ER)-positive HER2-negative breast cancer who underwent neoadjuvant therapy at Chiba University Hospital (Chiba, Japan) and Chiba Cancer Center (Chiba, Japan) from 2003 to 2015. We limited the inclusion criteria to postmenopausal patients with up to stage II. Patients with histories of systemic therapy for other cancers, including metachronous breast cancer, were excluded. Patients provided written informed consent for the use of their biological material for future research purposes before biopsy. The local ethics committee approved this study (Chiba University Hospital on 22 March 2017 No. 113. Chiba Cancer Center on 3 March 2017 No. 2285).

Traditional prognostic factors for early breast cancer including age at diagnosis, tumor size, lymph node (LN) involvement, and tumor grade were obtained from the electronic databases in each Facilities. The expression of ER, Progesterone receptor (PR), HER2, and Ki-67 was evaluated by immunohistochemistry in accordance with the ASCO/CAP guidelines [14, 15]. The cut off point for ER and PR positivity was 10% and that for Ki-67 positivity was 14%. HER2 was defined as positive with score 3 + , or demonstration of HER2 amplification on fluorescence in situ hybridization of ≧ 2.0.

The clinical response was assessed according to the response evaluation criteria in solid tumors (RECIST) guidelines [16]. For evaluating the pathological effect of neoadjuvant therapy, the response criteria of the Japanese Breast Cancer Society [17] was used. In order to evaluate the relationship between therapeutic effect and biological features, those who were pathological therapeutic effect grade 2 or 3 were defined as sensitive, and grade 0 or 1 as resistant.

The effects on prognosis were evaluated by recurrence-free survival (RFS) and overall survival (OS). RFS was defined as from the day systemic therapy started to the day of recurrence event (including locoregional recurrence, distant metastasis, and contralateral breast cancer). OS events were death from all causes.

### DNA extraction, High resolution melting analysis, and Sanger sequencing

Formalin fixed, paraffin embedded (FFPE) tissue samples of pretreatment core needle biopsy and surgical specimen were collected. The tumors were histologically evaluated on hematoxylin & eosin sections and we selected the area that contained more than 70% of the cancer cells. Four slices of 4-μm-thick section were made and then the tumor area of each section was manually dissected using a disposable scalpel. Total DNA was extracted from samples using the QIAamp DNA FFPE kit (Qiagen, Hilden, Germany) according to the manufacturer’s instructions. *PIK3CA* exons 9 and 20 were screened for mutations using high resolution melting (HRM) analysis on a LightCycler 480 (Roche Diagnostics, Mannheim, Germany) according to the protocol described before [18]. We used a plasmid containing mutation sequence for positive control. The primers used for analysis were shown in Supplementary Table S1. The M13 chimeric primers were used for subsequent Sanger sequencing.

*PIK3CA* (exons 9 and 20) polymerase chain reaction (PCR) products shown to be positive by HRM analysis were sequenced to confirm the presence of mutations using the BigDye Terminator v3.1 cycle sequencing kit (Applied Biosystems, Foster City, USA) according to the manufacturer's protocol. The M13 primers were used for sequencing. The sequencing products were analyzed on an ABI PRISM 3130 Genetic Analyzer (Applied Biosystems).

### Immunohistochemical analysis

Immunocytochemistry (IHC) was performed using ER (1D5; Dako, Tokyo, Japan) and PgR (PgR636; Dako), Ki-67(MIB-1; Dako), PTEN (6H2.1; Dako) antibodies and HercepTest II (Dako) following the manufacturer’s instructions. The stained slides were reviewed by two observers without knowledge of the clinical data. Staining as scored semi quantitatively using a histoscore (H-score), which was calculated as staining intensity (scored as 0–3) x percentage of stained cells (0–100%) as previously described [19]. Because the scoring system for PTEN expression by using immunohistochemical methods are not standardized, PTEN H-scores less than 50 were considered as low in this analysis,[20].

## Statistics

The association between various clinicopathological features and *PIK3CA* mutations or PTEN expression was compared using Fisher’s exact test or Wilcoxon’s test if appropriate. The probability of a pCR was estimated using a logistic regression model which was fitted to the pathological effects of neoadjuvant therapy, clinicopathological features, *PIK3CA* mutation status, and PTEN expression. In order to avoid overfitting, factors were carried in subsequent multivariate analyses if the statistical significance was *p* < 0.10 in univariate analyses. Odds ratios, 95% confidence intervals, and *p*-values were estimated. RFS and OS rates were calculated with the Kaplan–Meier method and evaluated with the log-rank test. Multivariate analyses of the predictors of pathological effects, recurrence, and death were conducted using the Cox proportional hazards model. All analyses were conducted with the JMP® software program (version 13.0, SAS Institute Inc., Cary, USA). *p*-values ≤ 0.05 were considered to indicate statistically significant differences.

## Results

### Patients’ characteristics

A total of 230 specimens were collected from 115 patients before and after treatment. Sixty patients underwent NAC including anthracyclines and taxanes. Fifty-five patients underwent NAE involving 6 months of aromatase inhibitor letrozole. The clinicopathological features of the patients collected from local medical records are shown in Table 1. In the NAE group, 40.0% of patients received standard adjuvant chemotherapy. In both groups, almost all patients received 5 years of adjuvant endocrine therapy. The median follow-up period was 72 (14–123) months in the NAC group and 68 (43–83) months in the NAE group.

**Table 1:** Clinicopathological characteristics of the patients

	**NAC (*n* = 60)**	**NAE (*n* = 55)**
Variables	No. of patients (%)	No. of patients (%)
Age at diagnosis, month median (range)	55 (45–73)	62 (52–75)
Tumor size		
T1	17 (28.3)	15 (27.3)
T2	43 (71.7)	40 (72.7)
LN involvement		
N0	11 (18.3)	55 (100.0)
N1	49 (81.7)	0 (0)
ER		
Positive	60 (100.0)	55 (100.0)
Negative	0 (0.0)	0 (0.0)
PR		
Positive	37 (61.7)	44 (80.0)
Negative	23 (38.8)	11 (20.0)
HER2		
Positive	0 (0.0)	0 (0.0)
Negative	60 (100.0)	55 (100.0)
Histological Grade		
1–2	46 (76.7)	52 (94.5)
3	14 (23.3)	3 (5.5)
Ki67 labeling Index		
< 14%	31 (51.7)	21 (38.2)
14%≦	29 (48.3)	34 (61.8)
Clinical Response		
PR / SD / PD	44 (73.3)	45 (81.8)
CR	16 (26.7)	10 (18.2)
Pathological therapeutic effect		
Grade 0	1 (1.7)	5 (9.1)
Grade 1	43 (71.7)	44 (80.0)
Grade2	13 (21.7)	5 (9.1)
Grade 3	3 (5.0)	1 (1.8)
Therapeutic effect for LN		
ypN0	28 (46.7)	48 (87.3)
ypN1	32 (53.3)	7 (12.7)
Adjuvant therapy^a^		
Chemotherapy	0 (0.0)	22 (40.0)
Endocrine therapy	57 (95.0)	55 (100.0)
Anti-HER2 therapy	2 (5.5)	0 (0.0)
Radiotherapy	39 (65.0)	50 (90.9)
Follow up period, month median (range)	72 (14–123)	68 (43–83)
Recurrence^a^		
All	16 (26.7)	0 (0.0)
Locoregional	7 (11.7)	-
Bone	3 (5.0)	-
Lung	4 (6.7)	-
Liver	3 (5.0)	-
others	3 (5.0)	-

*NAC* Neoadjuvant chemotherapy, *NAE* Neoadjuvant endocrine therapy, *LN* Lymph node, *ER* Estrogen receptor, *PR* Progesterone receptor, *HER2* Human epidermal growth factor receptor 2

^a^Including duplication

### *PIK3CA* mutations and PTEN expression

*PIK3CA* mutations were detected in 55 of pretreatment samples (47.8%) by HRM analysis and confirmed in 35 samples (30.4%) of these by Sanger sequence. These included 11 samples in exon 9, and 24 samples in exon 20 (Table 2). The mutation frequency was similar in each treatment group. Eight patients in the NAC group and 4 patients in the NAE group had no detectable mutations after treatment. Low PTEN expression by IHC was observed in 28 of pretreatment samples (24.3%; Table 2). Regardless of chemotherapy or endocrine therapy, both increased and decreased cases in PTEN expression were observed after treatment. There was no significant relationship between *PIK3CA* mutation status and PTEN expression (Supplementary Table S2). Clinicopathological features also had no significant relationship between *PIK3CA* mutations or PTEN expression (data not shown).

**Table 2:** *PIK3CA* mutation and PTEN expression in NAC and NAE patients

	**NAC (*n* = 60)**	**NAE (*n* = 55)**
	No. of patients (%)	No. of patients (%)
*PIK3CA* mutation		
All	19 (31.7)	16 (29.0)
Exon 9	8 (13.3)	3 (5.4)
Exon 20	11 (18.3)	13 (23.6)
PTEN expression		
Pretreatment		
Low	11 (18.3)	17 (30.9)
High	49 (81.7)	38 (69.1)
Posttreatment		
Low	18 (30.0)	13 (23.6)
High	39 (65.0)	41 (74.5)

### Pathological therapeutic response by *PIK3CA* mutation status and PTEN expression

Sixteen patients (26.7%) in the NAC group and 6 patients (10.9%) in the NAE group were judged as sensitive. In the NAC group, tumor with *PIK3CA* mutations showed significantly poorer response than tumor with *PIK3CA* wild-type (response rates 10.5% vs. 34.1%, *p* = 0.03). On the other hand, in the NAE group, there was no significant difference in pathological therapeutic effect between tumor with *PIK3CA* mutations and tumor with *PIK3CA* wild-type (response rates 6.3% vs. 12.9%, *p* = 0.54; Table 3). The response rates of NAC were 18.2% in PTEN low expression tumor and 28.5% in PTEN high expression tumor. The response rates of NAE were 5.9% in PTEN low expression tumor and 13.1% in PTEN high expression tumor. There was no association between PTEN expression and pathological therapeutic effect in both NAC and NAE group (*p* = 0.40 and *p* = 0.39, respectively; Table 4). In the NAC group, multivariate analysis revealed that only *PIK3CA* mutation status was significantly correlated with the pathological effect of NAC (Odds Ratio 5.26, *p* = 0.04; Table 5).

**Table 3:** Relationship between *PIK3CA* mutation and pathological therapeutic effect

	**NAC (*n* = 60)**			**NAE (*n* = 55)**		
	*PIK3CA* wild-type (*n* = 41)	*PIK3CA* mutation (*n* = 19)		*PIK3CA* wild-type (*n* = 39)	*PIK3CA* mutation (*n* = 16)	
	No. of patients (%)	No. of patients (%)	*p* value	No. of patients (%)	No. of patients (%)	*p* value
Therapeutic effect						
Grade 0	0 (0.0)	1 (5.3)		4 (10.3)	1 (6.3)	
Grade 1	27 (65.9)	16 (84.2)		30 (76.9)	14 (87.5)	
Grade 2	11 (26.8)	2 (10.5)		4 (10.3)	1 (6.3)	
Grade 3	3 (7.3)	0 (0.0)	**0.03**^a^	1 (2.6)	0(0.0)	0.54^a^

^a^Compared between Grade 0–1 and Grade 2–3

**Table 4:** Relationship between PTEN expression and pathological therapeutic effect

	**NAC (*n* = 60)**			**NAE (*n* = 55)**		
	PTEN low expression (*n* = 11)	PTEN high expression (*n* = 49)		PTEN low expression (*n* = 17)	PTEN high expression (*n* = 38)	
	No. of patients (%)	No. of patients (%)	*p* value	No. of patients (%)	No. of patients (%)	*p* value
Therapeutic effect						
Grade 0	0 (0.0)	1 (2.1)		0 (0.0)	5 (13.2)	
Grade 1	9 (81.8)	34 (69.4)		16 (94.1)	28 (73.7)	
Grade 2	2 (18.2)	11 (22.4)		1 (5.9)	4 (10.5)	
Grade 3	0 (0.0)	3 (6.1)	0.40^a^	0 (0.0)	1 (2.6)	0.39^a^

^a^Compared between Grade 0–1 and Grade 2–3

**Table 5:** Univariate and multivariate analysis for pathological effect of neoadjuvant chemotherapy

	**Univariate analysis**		**Multivariate analysis**	
Variables	Odds Ratio (95% CI)	*p* value	Odds Ratio (95% CI)	*p* value
Tumor size				
(T1 / T2)	0.71 (0.21–2.35)	0.57		
LN involvement				
(N0 / N1)	1.18 (0.27–5.06)	0.82		
PR				
(Positive/Negative)	2.78 (0.89–8.69)	0.07	2.67 (0.82–8.77)	0.10
Histological Grade				
(1–2 / 3)	2.12 (0.61–7.39)	0.24		
Ki67 labeling index				
(< 14 / ≧14)	1.10 (0.36–3.32)	0.87		
*PIK3CA* mutation				
(Mutant / Wild)	5.44 (1.11–26.78)	**0.04**	5.26 (1.04–26.51)	**0.04**
PTEN expression				
(Low / High)	2.18 (0.42–11.30)	0.35		

*LN* Lymph node, *PR* Progesterone receptor

### Prognosis by* PIK3CA* mutation status and PTEN expression

**Fig. 1:**
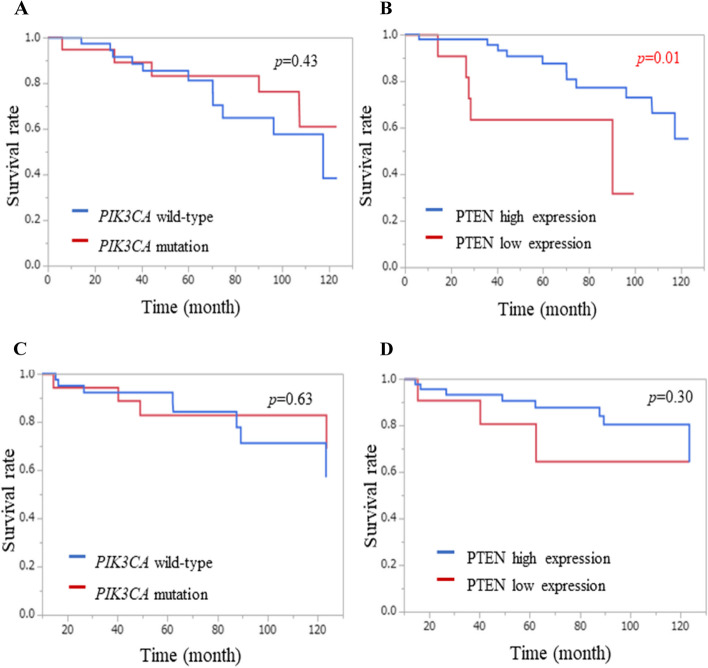
Kaplan–Meier curve for Recurrence-free survival (RFS; A and B) and Overall survival (OS; C and D). (**A**) RFS for patients with *PIK3CA* mutated and wild-type tumors; (**B**) RFS for patients with PTEN high and low expression tumors before treatment; (**C**) OS for patients with *PIK3CA* mutated and wild-type tumors; (**D**) OS for patients with PTEN high and low expression tumors before treatment

Further survival analyses were performed only in the NAC group because no recurrence was observed in the NAE group. In the NAC group, 5 patients (26.3%) with *PIK3CA* mutations and 11 patients (26.8%) with *PIK3CA* wild-type had RFS events. Four patients (21.1%) with *PIK3CA* mutations and 8 patients (19.5%) with *PIK3CA* wild-type died. Kaplan–Meier curves for RFS and OS in the NAC group are shown in Fig. 1. The log-rank test showed no difference in RFS between patients with *PIK3CA* mutations and *PIK3CA* wild-type tumors (Fig. 1A). In the NAC group, 5 patients (45.5%) with low PTEN expression and 11 patients (22.4%) with high PTEN expression had RFS events. Three patients (27.2%) with low PTEN expression and 9 patients (18.4%) with high PTEN expression died. Patients with low PTEN expression before chemotherapy showed significantly worse RFS compared to patients with high PTEN expression (5-year RFS 0.64 v 0.87, *p* = 0.01; Fig. 1B). There is no difference in OS between patients with *PIK3CA* mutations and *PIK3CA* wild-type (Fig. 1C), and between patients with high PTEN and low PTEN expression (Fig. 1D). In the Cox proportional hazards model for RFS, PTEN expression, PR, and therapeutic effect were predictive factors for time to recurrence (*p* < 0.01, *p* = 0.03, and *p* = 0.01, respectively; Table 6). The Cox proportional hazards model for OS showed no significant correlation between these factors and OS. The presence of *PIK3CA* mutations showed no effect on either RFS or OS (Table 6).

**Table 6:** Cox proportional hazards model for recurrence-free and overall survival of neoadjuvant chemotherapy

	**Recurrence-free survival**		**Overall survival**	
Variables	Hazard Ratio (95% CI)	*p* value	Hazard Ratio (95% CI)	*p* value
Tumor size				
(T1 / T2)	0.97 (0.22–3.72)	0.97	1.48 (0.32–6.07)	0.59
LN involvement				
(N0 / N1)	1.82 (0.47–6.03)	0.36	3.65 (0.96–13.15)	0.06
PR				
(Positive/Negative)	0.17 (0.04–0.61)	** < 0.01**	0.25 (0.06–1.03)	0.06
Histological Grade				
(1–2 / 3)	3.10 (0.55–59.18)	0.23	0.64 (0.14–3.74)	0.59
Ki67 labeling index				
(< 14 /≧14)	1.35 (0.38–4.97)	0.63	0.64 (0.17–2.35)	0.49
Therapeutic effect				
(Grade 0–1 / 2–3)	6.38 (1.15–5.96)	**0.03**	1.82 (0.37–10.60)	0.46
*PIK3CA* mutation				
(Mutant / Wild)	0.49 (0.13–1.62)	0.25	0.53 (0.10–2.35)	0.41
PTEN expression				
(Low / High)	6.38 (1.45–23.24)	**0.01**	2.71 (0.46–13.28)	0.25

*LN* Lymph node, *PR* Progesterone receptor

## Discussion

To our knowledge, this is the first report showing the effects of *PIK3CA* mutations and PTEN expression on pathological therapeutic effect of neoadjuvant therapy and the prognosis in postmenopausal Asian patients with ER-positive HER2-negative breast cancer. There are several treatment options for patients with early-stage luminal breast cancer, including surgery, chemotherapy, and endocrine therapy. Our study showed that in clinical practice, the examination of *PIK3CA* mutations and PTEN expression may provide useful information to determine the indication for neoadjuvant therapy.

Tumors with *PIK3CA* mutations had poorer response to NAC than tumors with *PIK3CA* wild-type. To confirm the effect of *PIK3CA* mutations on pathological therapeutic effect of NAC, multivariate analysis was performed. Only *PIK3CA* mutation status was an independent predictor for pathological therapeutic effect of NAC in postmenopausal luminal breast cancer patients. Although Ki-67 was reported as a predict marker of response to NAC even in luminal breast cancer [21], there is no relationship between them in our cohort. If postmenopausal patients with high Ki-67 luminal breast cancer plan NAC especially in expectation of tumor shrinkage in order to carry out breast conserving surgery, it would be better to confirm that tumor does not have *PIK3CA* mutations.

It has been reported that *PIK3CA* mutations are not related to the response to NAE in ER positive breast cancer [22, 23]. However, these studies included HER2-positive patients and some evaluation of the response was performed after short term treatment. Our cohort including only ER-positive, HER2-negative breast cancer patients also showed that *PIK3CA* mutations did not affect the response to 6 months NAE.

When response to NAC and prognosis were compared among breast cancer-intrinsic subtypes, patients with luminal tumors had a lower pathologic complete response rate but showed better outcomes compared with triple negative type and HER2 type [24]. Likewise, luminal tumors with *PIK3CA* mutations showed chemoresitance compared with *PIK3CA* wild-type but *PIK3CA* mutation status was not a prognostic marker for postmenopausal patients with luminal tumors. The relationship between *PIK3CA* mutations and prognosis in breast cancer patients is controversial. Kalinsky et al. [25] reported that patients with tumors harboring a *PIK3CA* mutation had significant improvement in OS and breast cancer-specific survival in 590 patients. A meta-analysis of eight retrospective cohort studies also showed that the group with *PIK3CA* mutation had superior clinical outcomes [26]. On the other hand, our study and a prospective clinical trial on adjuvant endocrine therapy [27] showed that *PIK3CA* mutations were not associated with prognosis. It is speculated that the impact of *PIK3CA* mutations on the prognosis varies with subtype and therapy, and there is no effect in postmenopausal luminal breast cancer patient.

Loss of PTEN expression had associations with poor prognosis and triple negative type in breast cancer patients as previously reported [28]. We confirmed that low PTEN expression before treatment is a poor prognostic factor even in luminal breast cancer. Expression changes in PTEN were seen after treatment (Table 2) but were not significantly associated with therapeutic effects in both NAC and NAE group (data not shown). There are various causes of loss of PTEN function, such as somatic mutation, epigenetic silencing, and protein interaction [29], and there are many ways to assess it. However, IHC may be a simple and useful test in the clinic to evaluate protein expression that reflects actual function of PTEN.

The frequency of *PIK3CA* mutations in patients with luminal breast cancer is 30–50% using digital PCR or next-generation sequencing (NGS) [1, 22, 23]. The Sanger sequence is less sensitive than PCR and NGS [30]. However, our study showed that the HRM analysis and Sanger sequencing method was useful for identifying clinically significant mutations. This method, which can be performed easily and inexpensively, has economic benefits given that *PIK3CA* mutations are clinically measured in over 1 million women with newly-diagnosed breast cancer, per year.

Our study had several limitations. First, DNA analysis using FFPE specimens is subject to DNA degradation that could affect the variability of the data. Second, this was a retrospective study; therefore, it is impossible to eliminate selection bias. Third, it is possible that our results were due to the small number of patients. Finally, our study only includes patients with ER-positive, HER2-negative breast cancer up to stage II, and it is unclear whether the findings would apply to patients with other subtypes or stages of breast cancer. The study only evaluates the relationship between PIK3CA mutations and PTEN expression with the response to neoadjuvant therapy and survival outcomes, and it is unclear whether these biomarkers have any therapeutic implications. Further prospective studies are needed to confirm our results.

## Conclusions

In conclusion, we found that *PIK3CA* mutations are associated with resistance to NAC but do not affect the response to NAE. We also found that low PTEN expression does not affect response to either NAC or NAE, but correlates with short RFS in patients who received NAC. Further evaluation of the potential for clinical use of these biomarkers in postmenopausal luminal breast cancer patients will be proposed following this study.

## Supplementary Information

**Additional file 1: Supplemental Table S1. **Primers for PCR and sequencing.**Supplementary Table S2.**Relationship between *PIK3CA* mutation and PTEN expression.

## Data Availability

The datasets generated during and/or analysed during the current study are available from the corresponding author on reasonable request.

## Abbreviations

NAC: Neoadjuvant chemotherapy
NAE: Neoadjuvant endocrine therapy
RFS: Recurrence-free survival
OS: Overall survival
PTEN: Phosphatase and tensin homolog
PIP2: Phosphatidylinositol bisphosphate
PIP3: Phosphatidylinositol triphosphate
ER: Estrogen receptor
PR: Progesterone receptor
HER2: Human epidermal growth factor receptor 2
TIL: Tumor-infiltrating lymphocytes
LN: Lymph node
RECIST: The response evaluation criteria in solid tumors
FFPE: Formalin fixed, paraffin embedded
HRM: High resolution melting
PCR: Polymerase chain reaction
IHC: Immunocytochemistry
H-score: Histoscore
NGS: Next-generation sequencing
