# Supplementary material for: The impact of PIK3CA mutations and PTEN expression on the effect of neoadjuvant therapy for postmenopausal luminal breast cancer patients
Source: BMC Cancer. 2023 Apr 27;23:384. doi: 10.1186/s12885-023-10853-y (PMC10134571; doi:10.1186/s12885-023-10853-y)
Supplement: Supplementary file 1 — Additional file 1: Supplemental Table S1. Primers for PCR and sequencing.Supplementary Table S2.Relationship between PIK3CA mutation and PTEN expression. [file 12885_2023_10853_MOESM1_ESM.docx]

Supplemental Table S1. Primers for PCR and sequencing

| Gene |  | Region | F/R | Seq (5´ > 3´) |
| --- | --- | --- | --- | --- |
| *PIK3CA* |  | Exon 9 | F | tgtaaaacgacggccagtGCAATTTCTACACGAGATCCTCT |
|  |  |  | R | caggaaacagctatgaccTTTAGCACTTACCTGTGACTCCA |
|  |  | Exon 20 | F | tgtaaaacgacggccagtCTGAGCAAGAGGCTTTGGAG |
|  |  |  | R | caggaaacagctatgaccTGTGTGGAAGATCCAATCCA |
|  | M13 sequences were shown in lower case. | | | |

Supplementary Table S2. Relationship between *PIK3CA* mutation and PTEN expression

|  | **NAC**  **(n=60)** | |  | **NAE**  **(n=55)** | |  |
| --- | --- | --- | --- | --- | --- | --- |
|  | *PIK3CA*  wild-type | *PIK3CA*  mutation |  | *PIK3CA*  wild-type | *PIK3CA*  mutation |  |
|  | No. of patients (%) | No. of patients (%) | *p* value | No. of patients (%) | No. of patients (%) | *p* value |
| PTEN expression |  |  |  |  |  |  |
| Low | 8 (19.5) | 3 (15.8) |  | 12(30.8) | 5 (31.3) |  |
| High | 33 (80.5) | 16 (84.2) | 0.52 | 27 (69.2) | 11 (68.7) | 0.60 |
